# Effects of Cystic Duct Margin Involvement on the Survival Rates of Patients With Gallbladder Cancer: A Propensity Score-Matched Case-Control Study

**DOI:** 10.7759/cureus.50585

**Published:** 2023-12-15

**Authors:** Vasistha Jajal, Phani K Nekarakanti, Sugumaran K, Hirdaya Nag

**Affiliations:** 1 Surgical Gastroenterology, Govind Ballabh Pant Institute of Postgraduate Medical Education and Research, New Delhi, IND

**Keywords:** gallbladder cancer, cancer survival, biliary surgery, frozen tissue biopsy, cystic duct margin

## Abstract

Background

In gallbladder cancer (GBC), extrahepatic bile duct (EHBD) resection is selectively performed if gross direct extension or microscopic involvement of the cystic duct margin (CDM) is detected. Although CDM is usually sent for frozen biopsy intraoperatively in most centers, there are no studies regarding the routine use of CDM frozen biopsy irrespective of the tumor location and paucity of literature regarding the impact of CDM status on recurrence-free and overall survival in GBC. The presence of obstructive jaundice in GBC usually indicates the involvement of EHBD or cystic duct-bile duct junction. The present study aimed to analyze the necessity of routine CDM frozen biopsy in patients with resectable GBC without jaundice, regardless of the tumor location. The impact of positive CDM on survival was also evaluated.

Methods

This retrospective observational case-control study was conducted from May 2009 to March 2021 and included 105 patients with resectable GBC without macroscopic EHBD infiltration and jaundice. Patients were divided into CDM-negative (n=91) and CDM-positive (n=14) groups. Propensity score matching was performed for variables such as performance status, tumor size, tumor-node-metastasis (TNM) stage, and adjuvant chemotherapy. After propensity score matching, 27 patients (CDM-negative=13, CDM-positive=14) were included. The primary outcome was to analyze the role of routine CDM frozen biopsy regardless of tumor location, and secondary outcomes were to study the impact of positive CDM status on survival and evaluate predictive factors for CDM positivity. A subgroup analysis was conducted to assess clinicopathologic characteristics and outcomes of the anatomical location of the tumor.

Results

Of 105 patients, 91 had negative CDM, and 14 had positive CDM. Among 14 patients with positive CDM, only one patient had a tumor in the fundus/body, and the remaining had a tumor involving the neck. All CDM-positive patients underwent bile duct excision with hepaticojejunostomy. Common bile duct (CBD) involvement was present in 50% of patients with positive CDM in the final histopathological examination. In the matched population, patients with positive CDM had a significantly higher rate of neck tumors (p=0.001). Recurrence-free survival (24 vs. 12 months, p=0.30) and overall survival (24.5 vs. 20 months, p=0.417) were comparable between CDM-negative and CDM-positive groups, respectively. On multivariate analysis, preoperative and intraoperative tumor location were independent predictive factors for CDM positivity. On subgroup analysis, 30 patients had tumor involving the neck of the gallbladder, and the remaining 75 had at the fundus and body of the gallbladder. Neck tumors had inferior recurrence-free survival (17 vs. 30 months, p=0.012) and overall survival (24 vs. 36 months, p=0.048) compared to non-neck tumors.

Conclusions

Routine use of CDM frozen analysis in patients with resectable GBC without jaundice, regardless of tumor location, can be avoided. It can be selectively preferred in patients with GBC involving the neck since tumor location is found to be an independent predictive factor for CDM positivity. Positive CDM has comparable survival outcomes to negative CDM, providing a similar R0 resection rate and tumor stage. However, neck tumors have a worse prognosis than non-neck tumors.

## Introduction

Gallbladder cancer (GBC) is a very aggressive tumor with a dismal outcome, with a mean overall survival (OS) of six months and a five-year OS rate of 5% [1]. Approximately 60% of the patients with GBC have tumor in the fundus, 30% in the body, and 10% in the neck [2]. Seventy-five percent of the patients have unresectable or metastatic disease and are not candidates for curative resection [3]. However, the prognosis after surgical resection remains poor, with a five-year survival rate between 10% and 90% depending on disease factors such as tumor grade and stage [4]. The standard surgical procedure for GBC is radical cholecystectomy with or without bile duct resection. Routine extrahepatic bile duct (EHBD) resection, previously recommended for nodal clearance, is now performed selectively to achieve negative margins, as studies have shown no impact on survival but increased morbidity [4-7]. The bile duct resection is only performed if gross direct extension or microscopic involvement of the cystic duct margin (CDM) is examined intraoperatively [3]. Patients with macroscopic infiltration of hepatoduodenal ligament (HDL) or EHBD often present with obstructive jaundice [8]. Though the CDM is usually sent for frozen biopsy during surgery for GBC in most centers, there are no studies in the literature regarding its routine use irrespective of the tumor location.

Positive CDM during index cholecystectomy in incidental gallbladder cancer (IGBC) is a significant negative prognostic factor [9]. Patients with positive CDM in IGBC are more likely to have residual disease in the EHBD (positive cystic duct, 42.1% vs. negative cystic duct, 4.3%) [10]. No studies demonstrate the impact of positive CDM on survival outcomes in resectable GBC. Thus, in our study, we aim to analyze the role of routine CDM frozen section in resectable GBC without macroscopic EHBD infiltration and jaundice regardless of the tumor location and its impact on survival, to evaluate predictive factors for CDM positivity, and also to assess clinicopathologic characteristics and outcomes of the anatomical location of the tumor in GBC.

## Materials and methods

**Figure 1:**
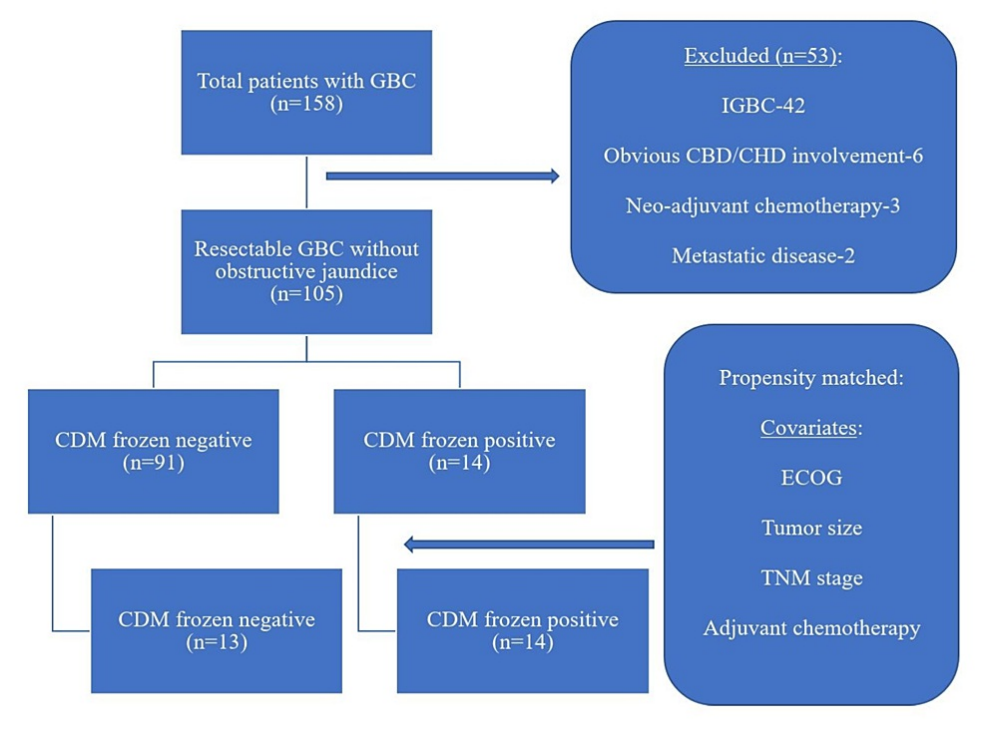
Flowchart showing the selection of the study population. GBC: gallbladder cancer; IGBC: incidental gallbladder cancer; CBD: common bile duct; CHD: common hepatic duct; CDM: cystic duct margin; ECOG: Eastern Cooperative Oncology Group; TNM: tumor-node-metastasis

This was a retrospective observational case-control study conducted at Govind Ballabh Pant Institute of Postgraduate Medical Education and Research, Maulana Azad Medical College, Delhi University, New Delhi, India. A total of 158 patients diagnosed with operable GBC were treated from May 2009 to March 2021. The study included patients with resectable GBC without clinical obstructive jaundice (n=105). Patients with IGBC (n=42), patients with obvious common hepatic duct (CHD)/common bile duct (CBD) involvement diagnosed preoperatively or intraoperatively (n=6), patients who received neoadjuvant therapy (n=3), and patients with metastatic disease diagnosed intraoperatively (M1, n=2) were excluded from the study (n=53). Patients were divided into two groups based on frozen section analysis: (1) CDM-negative and (2) CDM-positive. Propensity score matching (PSM) and analysis were performed for variables such as Eastern Cooperative Oncology Group (ECOG) performance status, tumor size, tumor-node-metastasis (TNM) stage, and adjuvant chemotherapy (Figure 1).

Outcomes

The primary outcome was to analyze the role of routine frozen biopsy of CDM in resectable GBC without obstructive jaundice. Secondary outcomes were to analyze the impact of positive CDM status on survival, to evaluate preoperative and intraoperative predictive factors for CDM positivity, and to evaluate clinicopathologic characteristics and outcomes of the anatomical location of the tumor.

Postoperative complications were graded according to the Clavien-Dindo classification system [11]. The TNM staging of GBC was based on the 8th edition of the American Joint Committee on Cancer (AJCC) [12]. OS was defined as the time from surgery to death from any cause or last follow‐up (censored). Recurrence-free survival (RFS) was defined as the time from surgery to recurrence (diagnosed clinically and/or by imaging).

Definition of tumor location

The gallbladder (GB) is anatomically divided into fundus, body, infundibulum, and neck [13]. The neck is defined as the portion between the infundibulum and the cystic duct. Patients in the same study cohort were divided into two groups for subgroup analysis based on tumor involvement: (1) GBC with neck involvement and (2) GBC not involving the neck. The first group included patients with tumors involving the neck (e.g., tumor in the neck, tumor in the body and neck, and tumor replacing the GB and involving the neck). The second group included patients with tumors not involving the neck (e.g., tumor in the fundus, tumor in the body, and tumor in the fundus and the body). Histologically, the neck was defined as the area adjacent to the cystic duct having tubulo-alveolar mucus glands [14]. The fundus/body region was defined as the region distal to the neck where mucus glands are absent [14].

Management protocol

All patients with GBC underwent clinical examination, followed by routine blood investigations with tumor markers such as carbohydrate antigen 19-9 (CA 19-9) and carcinoembryonic antigen (CEA), ultrasound (USG) of the abdomen, and contrast-enhanced computed tomography (CECT) of the chest and abdomen. The tumor site and extent have been defined by the CECT abdomen. Endoscopic ultrasound (EUS) with fine needle aspiration cytology (FNAC) was performed selectively in patients with enlarged inter-aortocaval (IAC) or para-aortic lymph nodes diagnosed on CECT abdomen. Patients with positive EUS-FNAC were referred for definitive chemotherapy and excluded from the study. Patients with resectable GBC were posted for surgical resection.

Surgical procedure

Staging laparoscopy was performed in patients with resectable disease. Patients with intraoperatively diagnosed metastatic disease were excluded from the study. Patients without evidence of metastasis underwent further assessment for resectability. If resectable, radical cholecystectomy was performed, including en bloc cholecystectomy with either anatomical segment 4b and 5 resection or non-anatomical 2 cm wedge resection and lymphadenectomy (involving stations 8, 12, and 13). Resections were performed laparoscopically whenever feasible, as described by Nag et al. [15,16]. Conversion to open laparotomy was performed whenever required (e.g., technical difficulty, intraoperative bleeding, and multi-visceral resection).

CDM frozen section analysis was performed in all patients regardless of tumor location. According to CDM frozen status, patients were divided into CDM-positive and CDM-negative groups. In patients with positive CDM, EHBD excision with Roux-en-Y hepaticojejunostomy (RYHJ) was performed. Those with adjacent organ involvement underwent multi-visceral resections. If unresectable, then patients were planned for palliative chemotherapy.

Follow-up

Patients were followed up in the outpatient department (OPD) every three months with history, physical examination, CEA and CA 19-9, and USG of the abdomen in the first year and then every six months after that. CECT abdomen was carried out every six months during the first two years and then annually. After a multidisciplinary tumor board discussion, patients were planned for adjuvant chemotherapy based on histopathological TNM stage (>T2 and/or N+ disease). Recurrence was diagnosed either clinically or radiologically.

Statistical analysis

Data processing was performed using IBM SPSS Statistics for Windows, Version 26.0 (Released 2019; IBM Corp., Armonk, New York, United States). Data were described using range, mean±standard deviation, median, frequencies (number of cases), and relative frequencies (percentages) as appropriate. To determine whether the data were normally distributed, a Kolmogorov-Smirnov test was used. Comparison of quantitative variables between study groups was performed using the Student t test and Mann-Whitney U test for parametric and non-parametric data, respectively. Chi-squared test was performed to compare categorical data, and Fisher's exact test was performed when the expected frequency was less than five. Survival curves were constructed using the Kaplan-Meier method and compared using the log-rank test. We performed Cox regression analysis to identify the preoperative and intraoperative predictive factors for CDM positivity. Variables found to be significant in the univariate analysis were also included in the multivariate analysis. A p-value (two-sided) of <0.05 is considered statistically significant.

## Results

A total of 14 patients (13.3%) had positive CDM among 105 resectable GBC patients. Patients were divided into CDM-positive (n=14) and CDM-negative (n=91) in the unmatched study population. After PSM, 27 patients (CDM-positive=14, CDM-negative=13) were included in the analysis. A comparative subgroup analysis was performed between patients with GB neck tumors (n=30) and GB fundus/body tumors (n=75) in the same study population (N=105) to evaluate clinicopathologic characteristics and outcomes of the anatomical location of the tumor.

Unmatched cohort

In the unmatched study population, baseline demographics were comparable between the two groups, except for a higher ECOG performance status in the CDM-positive patients. Patients with CDM positivity had a higher rate of cholangitis and a higher rate of tumors involving the neck of the GB on CT. Patients with CDM positivity had a significantly longer duration of surgery, greater intraoperative blood loss, and higher morbidity (higher Clavien-Dindo grade of complications). All patients with CDM positivity underwent CBD excision with RYHJ. One patient with negative CDM underwent CBD excision with RYHJ due to a lymph node mass encasing CBD (Table 1).

**Table 1:** Baseline clinical characteristics. CDM: cystic duct margin; ECOG: Eastern Cooperative Oncology Group; CT: computed tomography; TLC: total leucocyte count; AST: aspartate aminotransferase; ALT: alanine aminotransferase; ALP: alkaline phosphatase; PT INR: prothrombin time international normalized ratio; CEA: carcinoma embryonic antigen; CA 19-9: carbohydrate antigen 19-9; GB: gallbladder; LN: lymph node, CBD: common bile duct; ECB: extended cholecystectomy involving bi-segmentectomy segment 4b and 5; ECW: extended cholecystectomy involving wedge hepatic resection; CBDE: CBD excision; RYHJ: Roux-en-Y hepaticojejunostomy; GJ: gastrojejunostomy; RA: resection anastomosis; CD: Clavien-Dindo; -: not available *: values expressed in median (interquartile range); **: values expressed in n (%), #: statistically significant (p<0.05)

Variables	Before matching			After matching		
	CDM-negative (n=91)	CDM-positive (n=14)	p-value	CDM-negative (n=13)	CDM-positive (n=14)	p-value
Age in years*	50 (42-60)	57 (45-60)	0.260	52 (43-56)	57 (45-60)	0.368
Gender**			0.441			0.308
Female	58 (63.8)	11 (78.6)		8 (61.5)	11 (78.6)	
Comorbidity**	15 (16.4)	3 (21.4)	0.648	2 (15.4)	3 (21.4)	0.686
ECOG**			0.011^#^			0.842
0	12 (13.1)	0 (0)		0 (0)	0 (0)	
1	64 (70.3)	7 (50)		7 (53.8)	7 (50)	
2	15 (16.4)	7 (50)		6 (46.2)	7 (50)	
3	0 (0)	0 (0)		0 (0)	0 (0)	
Presenting complaints**						
Pain	87 (95.6)	12 (85.7)	0.137	13 (100)	12 (85.7)	0.541
Cholangitis	1 (1.1)	2 (14.3)	0.005^#^	0 (0)	2 (14.3)	0.481
Dyspepsia	3 (3.3)	0 (0)	0.491	0 (0)	0 (0)	-
Location of mass on imaging (CT)**			0.001^#^			0.001^#^
Neck involved	17 (18.7)	13 (92.9)		2 (15.4)	13 (92.9)	
Neck not involved	74 (81.3)	1 (7.1)		11 (84.6)	1 (7.1)	
Biochemical parameters*						
Hemoglobin, g/dL	11 (9.8-12.2)	10.1 (9.5-11.7)	0.258	11.1 (10-12.05)	10.1 (9.5-11.7)	0.233
TLC, cells/m^3^	8.5 (7.1-10.4)	9.05 (6.3-12.8)	0.353	8.8 (6.9-11.4)	9.05 (6.3-12.8)	0.923
Platelets, x10^5^ cells/mL	230 (187-300)	210 (174-245)	0.258	210.5 (178-250)	210 (174-245)	0.497
Bilirubin, mg/dL	0.7 (0.5-0.9)	0.8 (0.5-1.8)	0.775	0.6 (0.5-0.8)	0.8 (0.5-1.8)	0.203
AST, IU/L	38 (27-67)	50 (32-66)	0.766	32 (24.5-48)	50 (32-66)	0.114
ALT, IU/L	36 (28-85)	35 (26-64)	0.084	42 (17.5-63)	35 (26-64)	0.903
ALP, IU/L	111 (87-189)	160 (126-245)	0.054	110 (85.5-204.5)	160 (126-245)	0.069
PT INR	1.08 (1-1.13)	1.06 (1-1.14)	0.684	1.1 (1-1.23)	1.06 (1-1.14)	0.696
Tumor markers*						
CEA, ng/mL	2.1 (1.2-4)	2.2 (1.1-5.5)	0.553	2.8 (1.2-5.65)	2.2 (1.1-5.5)	0.789
CA 19-9, IU/mL	57.6 (7.8-134)	77.0 (9.5-151.3)	0.184	64.0 (7.6-146.8)	77.0 (9.5-151.3)	0.716
Lap**	23 (25.2)	0 (0)	0.036^#^	5 (38.5)	0 (0)	0.016^#^
Operative findings**						
GB mass with liver infiltration	34 (37.4)	10 (71.4)	0.016^#^	8 (61.5)	10 (71.4)	0.695
Duodenal infiltration	7 (7.7)	1 (7.1)	0.942	0 (0)	1 (7.1)	0.326
Colon infiltration	1 (1.1)	1 (7.1)	0.744	2 (15.4)	1 (7.1)	0.496
LN mass, encasing CBD	1 (1.1)	0 (0)	0.693	0 (0)	0 (0)	-
Intra-op location of mass**			0.001^#^			0.001^#^
Involving neck	12 (13.2)	12 (85.7)		2 (15.4)	12 (85.7)	
Type of surgery**						
ECB	50 (54.9)	8 (57.1)	0.878	6 (46.2)	8 (57.1)	0.706
ECW	41 (45.1)	6 (42.9)	0.878	7 (53.8)	6 (42.9)	0.706
Additional (adjacent organ) resection**						
CBDE with RYHJ	1 (1.1)	14 (100)	0.001^#^	0 (0)	14 (100)	0.001^#^
Distal gastrectomy with duodenectomy with GJ	7 (7.7)	1 (7.1)	0.942	0 (0)	1 (7.1)	0.326
Duodenal sleeve resection	0 (0)	0 (0)	-	0 (0)	0 (0)	-
Colonic RA	5 (5.5)	0 (0)	0.369	1 (7.7)	0 (0)	0.481
Colonic sleeve resection	3 (3.3)	1 (7.1)	0.484	1 (7.7)	1 (7.1)	0.958
Surgery duration, in minutes*	240 (200-320)	330 (240-422)	0.008^#^	300 (210-360)	330 (240-422)	0.341
Intra-op blood loss, in mL*	180 (120-240)	375 (200-425)	0.000^#^	200 (165-275)	375 (200-425)	0.026^#^
Morbidity and mortality**			0.020^#^			0.722
CD1	76 (83.5)	7 (50)		7 (53.8)	7 (50)	
CD2	11 (12.1)	5 (35.7)		4 (30.8)	5 (35.7)	
CD3	2 (2.2)	1 (7.1)		1 (7.7)	1 (7.1)	
CD4	1 (1.1)	1 (7.1)		0 (0)	1 (7.1)	
CD5	1 (1.1)	0 (0)		1 (7.7)	0 (0)	
In-hospital mortality**	1 (1.1)	0 (0)	0.693	1 (7.7)	0 (0)	0.133

**Figure 2:**
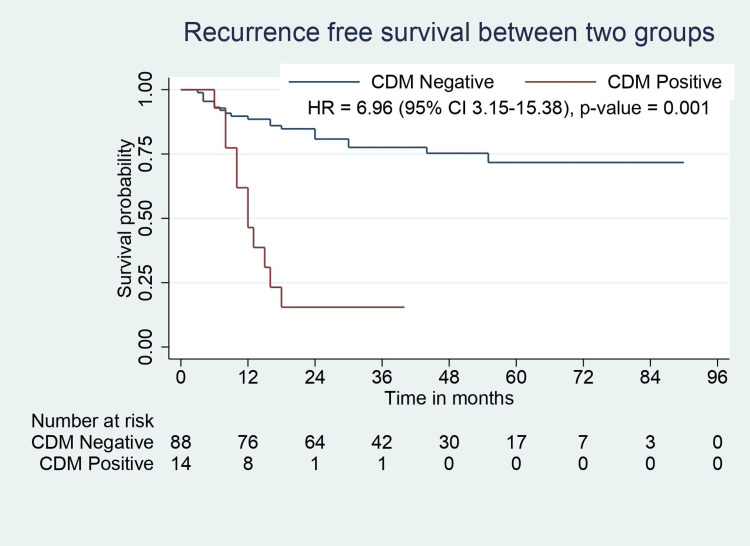
RFS analysis for CDM-negative and CDM-positive groups. RFS: recurrence-free survival; CDM: cystic duct margin; CI: confidence interval; HR: hazard ratio

**Figure 3:**
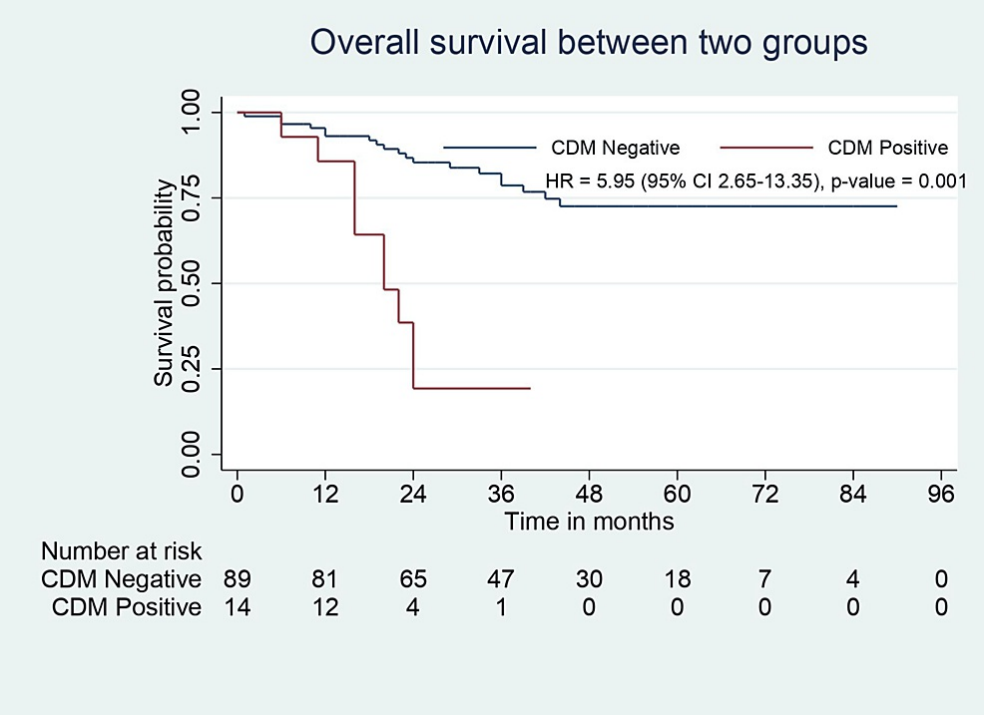
OS analysis for CDM-negative and CDM-positive groups. OS: overall survival; CDM: cystic duct margin; CI: confidence interval; HR: hazard ratio

All patients in this study population had negative resection margins (R0). Pathological features such as perineural involvement, number of positive lymph nodes, liver involvement, and CBD involvement were significantly higher in CDM-positive patients. CDM-positive patients had higher tumor stage but were not statistically significant (p=0.055). CBD involvement was present in 50% of patients with positive CDM. One of 14 patients with positive CDM had a tumor that did not involve the neck region (with fundus/body involvement). Although the recurrence rate was higher in CDM-positive patients, the recurrence sites were similar between the groups. Median RFS (31.5 months vs. 12 months, p=0.001) and OS (36 months vs. 20 months, p=0.001) were significantly lower in CDM-positive patients (Table 2, Figure 2, Figure 3).

**Table 2:** Pathological data and outcome. LVI: lymphovascular invasion; PNI: perineural invasion; CDM: cystic duct margin; WD: well differentiated; MD: moderately differentiated; PD: poorly differentiated; LN: lymph node; TNM: tumor-node-metastasis; AJCC: American Joint Committee on Cancer; CBD: common bile duct; R1: microscopic residual tumor; RFS: recurrence-free survival; OS: overall survival; -: not available *: values expressed in median (interquartile range); **: values expressed in n (%); #: statistically significant (p<0.05)

Variable	Before matching			After matching		
	CDM-negative (n=91)	CDM-positive (n=14)	p-value	CDM-negative (n=13)	CDM-positive (n=14)	p-value
Tumor grade**			0.285			0.603
WD	23 (25.3)	1 (7.1)		1 (7.7)	1 (7.1)	
MD	56 (61.5)	10 (71.4)		11 (84.6)	10 (71.4)	
PD	12 (13.2)	3 (21.4)		1 (7.7)	3 (21.4)	
Tumor site**			0.001^#^			0.001^#^
Involving neck	17 (18.7)	13 (92.9)		2 (15.4)	13 (92.9)	
Tumor size, in cm*	3.0 (2.0-4.2)	3.5 (2.5-5.2)	0.436	3.0 (2-4.6)	3.5 (2.5-5.2)	0.477
LVI**	21 (23.1)	3 (21.4)	0.891	7 (53.8)	3 (21.4)	0.120
PNI**	25 (27.5)	9 (64.3)	0.006^#^	7 (53.8)	9 (64.3)	0.704
Both invasion (LVI+PNI)**	14 (15.4)	3 (21.4)	0.568	6 (46.2)	3 (21.4)	0.236
Total LN retrieved*	12 (9-15)	16 (10-21)	0.014^#^	10 (8.5-16.5)	14.5 (10.75-21.5)	0.093
Positive LN*	1.5 (0-6.5)	3 (1.5-5)	0.005^#^	4 (0.5-4)	3 (1.5-5)	0.940
TNM stage (AJCC 8th edition)**			0.055			0.695
I	14 (15.4)	0 (0)		0 (0)	0 (0)	
II	30 (32.9)	1 (7.1)		1 (7.7)	1 (7.1)	
III	33 (36.3)	6 (42.9)		6 (46.2)	6 (42.9)	
IV	14 (15.4)	7 (50)		6 (46.2)	7 (50)	
Liver involvement**	22 (24.2)	11 (78.6)	0.001^#^	7 (53.8)	11 (78.6)	0.236
CBD involvement**	0 (0)	7 (50)	0.001^#^	0 (0)	7 (50)	0.006^#^
Duodenum/stomach involvement**	13 (14.3)	1 (7.1)	0.369	0 (0)	1 (7.1)	0.326
Colon involvement**	6 (6.6)	1 (7.1)	0.939	0 (0)	1 (7.1)	0.326
Hospital stay, in days*	6.0 (5-9.0)	11.5 (7.75-15.5)	0.002^#^	5 (4-7)	11.5 (7.75-15.5)	0.002^#^
Adjuvant chemotherapy**	46 (50.5)	10 (71.4)	0.145	9 (69.2)	10 (71.4)	0.901
Recurrence**						
Yes	20 (21.9)	11 (78.6)	0.001^#^	6 (46.2)	11 (78.6)	0.389
Not known (lost to follow-up)	5 (5.5)	1 (7.1)	0.921	2 (15.4)	1 (7.1)	0.277
Site of recurrence**			0.568			0.701
Liver	7 (35)	3 (27.3)		1 (16.67)	3 (27.3)	
Hilum	4 (20)	3 (27.3)		1 (16.67)	3 (27.3)	
Locoregional LN	4 (20)	4 (36.3)		2 (33.3)	4 (36.3)	
Distant	5 (25)	1 (9.1)		2 (33.3)	1 (9.1)	
RFS, in months*	31.5 (20-54)	12 (8-16.5)	0.001^#^	24 (7-52)	12 (8-16.5)	0.300
OS, in months*	36 (22-54)	20 (16-24)	0.001^#^	24.5 (8-47)	20 (16-24)	0.417

Matched cohort

**Figure 4:**
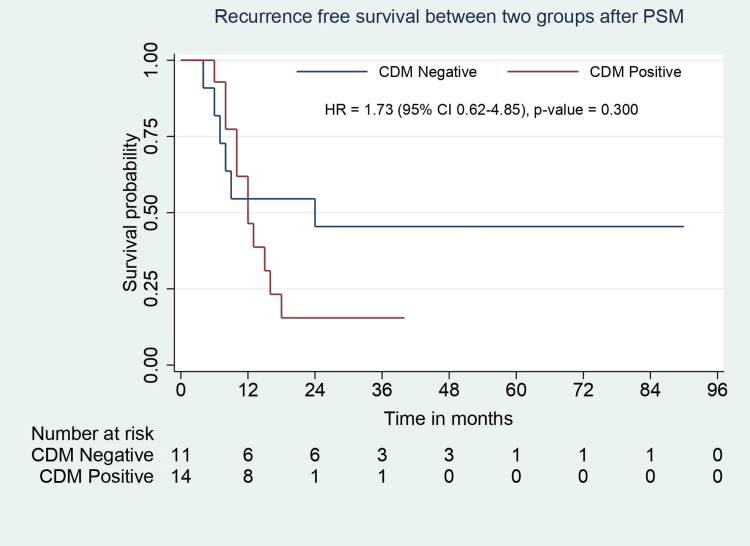
RFS analysis for CDM-negative and CDM-positive groups after PSM. RFS: recurrence-free survival; PSM: propensity score matching; CDM: cystic duct margin; CI: confidence interval; HR: hazard ratio

**Figure 5:**
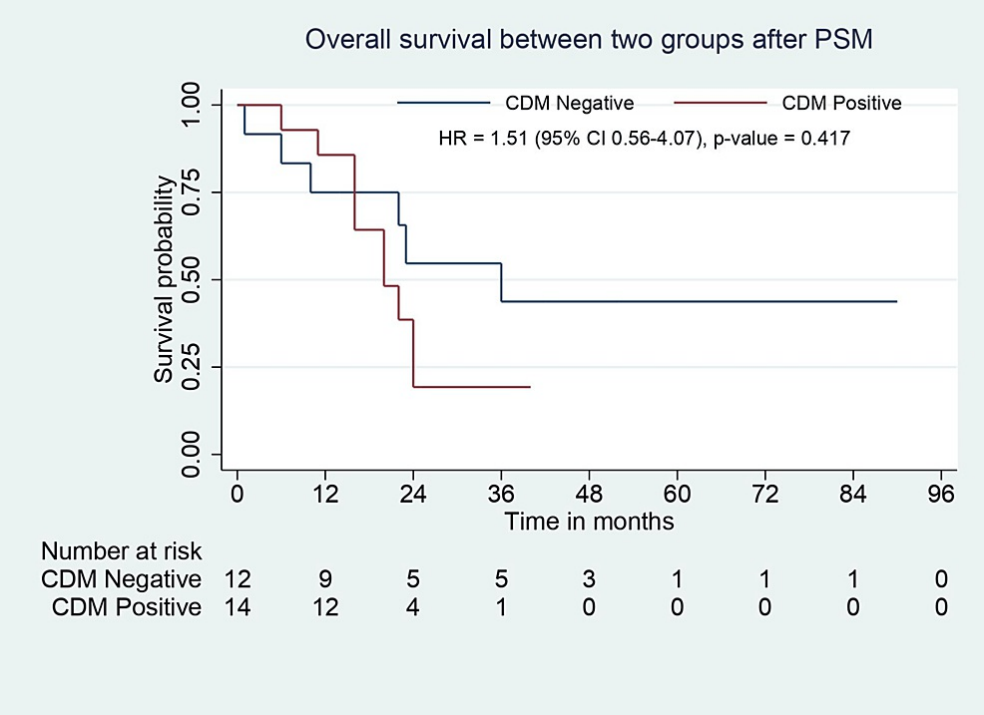
OS analysis for CDM-negative and CDM-positive groups after PSM. OS: overall survival; PSM: propensity score matching; CDM: cystic duct margin; CI: confidence interval; HR: hazard ratio

In the matched study population, the baseline demographic, clinical, and biochemical parameters were comparable between groups except for tumor location on CT. Patients with positive CDM had a significantly higher rate of neck tumors (p=0.001). The types of surgeries performed were similar in the groups, except patients with CDM positivity required an additional CBD excision with RYHJ (p=0.001). The operative time was comparable, but intraoperative blood loss was significantly higher in CDM-positive patients (p=0.026). The distribution of Clavien-Dindo complication grades was similar between groups (Table 1). Pathological features such as tumor type, tumor grade, lymphovascular invasion (LVI), perineural invasion (PNI), number of positive lymph nodes, and TNM stage were comparable except for tumor location. Patients with CDM positivity had a longer hospital stay (p=0.002). The groups were comparable in terms of adjuvant chemotherapy, recurrence rate, and site of recurrence. Median RFS (24 months vs. 12 months, p=0.30) and OS (24.5 months vs. 20 months, p=0.417) were comparable between groups (Table 2, Figure 4, Figure 5).

Predictive factors for CDM positivity

In univariate analysis, the presence of cholangitis, the location of the mass on CT, regional lymphadenopathy and IHBRD on CT, the intraoperative location of the mass, and liver infiltration were significant factors predicting CDM positivity. On multivariate analysis, preoperative location of mass on CT and intraoperative location of mass were independent predictive factors for CDM positivity (Table 3).

**Table 3:** Univariate and multivariate analysis of preoperative and intraoperative predictive factors for CDM positivity. CDM: cystic duct margin; CI: confidence interval; CT: computed tomography; IHBRD: intra-hepatic biliary radicle dilatation; ALP: alkaline phosphatase; -: not significant #: statistically significant (p<0.05)

Variable	Univariate analysis p-value	Multivariate analysis		
		Hazard ratio	95% CI	p-value
Preoperative factors:				
Complaints				
Jaundice	0.072	-	-	-
Cholangitis	0.005^#^	-	-	-
On imaging (CT)				
Location of mass (involving the neck)	0.001^#^	51.14	2.405-1087.844	0.012^#^
Liver infiltration	0.067	-	-	-
Peri-portal/peri-pancreatic lymphadenopathy	0.001^#^	-	-	-
IHBRD	0.009^#^	-	-	-
Biochemical parameter				
ALP	0.054	-	-	-
Intraoperative factors:				
Intra-op location of mass (involving the neck)	0.001^#^	52.53	1.784-1546.767	0.022^#^
Liver infiltration	0.016^#^	-	-	-
Peri-portal/peri-pancreatic lymphadenopathy	0.805	-	-	-

Subgroup analysis

A comparative subgroup analysis was performed between patients with GB neck tumors (n=30) and without GB neck tumors (n=75) in the same study population (N=105). Patients with neck tumors had higher bilirubin and alkaline phosphatase (ALP) levels preoperatively. 43.3% of patients with tumor in the neck region and 1.33% of patients with tumors without neck involvement had a positive CDM. One of 14 (7.14%) patients with positive CDM had a tumor in the fundus/body region, and the rest involved the neck. Intraoperatively, tumors involving the neck had higher CDM positivity and longer operative time. Patients with neck tumors had a higher T stage (p=0.011), a higher rate of the liver (p=0.001) and CBD involvement (p=0.002), and a higher recurrence rate (p=0.002). Median RFS (30 months vs. 17 months, p=0.012) and OS (36 months vs. 24 months, p=0.048) were significantly lower in neck tumors (Table 4).

**Table 4:** Subgroup analysis between GBC involving the neck and GBC not involving the neck. CDM: cystic duct margin; LVI: lymphovascular invasion; PNI: perineural invasion; GBC: gallbladder cancer; ALP: alkaline phosphatase; CA 19-9: carbohydrate antigen 19-9; WD: well differentiated; MD: moderately differentiated; PD: poorly differentiated; TNM: tumor-node-metastasis; AJCC: American Joint Committee on Cancer; CBD: common bile duct; LN: lymph node; RFS: recurrence-free survival; OS: overall survival *: values expressed in median (interquartile range); **: values expressed in n (%), #: statistically significant (p<0.05)

Variables	GBC not involving the neck (n=75)	GBC involving the neck (n=30)	p-value
Preoperative variables:			
Bilirubin*	0.6 (0.5-0.9)	0.9 (0.5-1.7)	0.032^#^
ALP*	110 (87-153)	160 (104-237)	0.007^#^
CA 19-9*	17.9 (8.4-34)	21.1 (7-35.5)	0.975
Intraoperative variables:			
Positive CDM frozen**	1 (1.3)	13 (43.3)	0.001^#^
Surgery duration*	240 (190-300)	300 (230-400)	0.034^#^
Pathologic data and outcome:			
Histologic grade**			0.114
WD	21 (28.0)	3 (10.0)	
MD	45 (60.0)	21 (70.0)	
PD	9 (12.0)	6 (20.0)	
LVI**	16 (21.3)	8 (26.7)	0.610
PNI**	19 (25.3)	15 (50.0)	0.021^#^
Both invasion (LVI+PNI)**	9 (12.0)	8 (26.7)	0.081
TNM stage (AJCC 8th edition)**			0.086
T stage**			0.011^#^
T1b	13 (17.4)	2 (6.7)	
T2a	12 (16.0)	2 (6.7)	
T2b	24 (32.0)	7 (23.3)	
T3	26 (34.7)	18 (60)	
T4	0 (0)	1 (3.3)	
N stage**			0.089
N0	51 (68.0)	15 (50.0)	
N1	13 (17.3)	5 (16.7)	
N2	11 (14.7)	10 (33.3)	
Liver involvement**	16 (21.3)	17 (56.7)	0.001^#^
CBD involvement**	1 (1.3)	6 (20.0)	0.002^#^
Adjuvant chemotherapy**	37 (49.3)	19 (63.3)	0.279
Recurrence**	15 (20)	14 (46.7)	0.002^#^
Site of recurrence**			0.122
Liver	4 (26.67)	5 (35.7)	
Hilum	4 (26.67)	3 (21.4)	
Locoregional LN	4 (26.67)	3 (21.4)	
Distant	3 (20.0)	3 (21.4)	
RFS, in months*	30 (20-54)	17 (8-41)	0.012^#^
OS, in months*	36 (22-54)	24 (16-41)	0.048^#^

## Discussion

In GBC, curative resection with an R0 resection margin remains the best chance for long-term survival. Resection of the EHBD in GBC should be selective and reserved only for patients with gross involvement by direct infiltration or microscopic involvement of the intraoperatively assessed frozen status of the CDM and HDL lymph nodes densely adherent to EHBD. Since there is no standardized protocol regarding intraoperative CDM frozen sections, some centers routinely use CDM frozen biopsy, while others do not due to the unavailability of frozen sections and analysis. There are no studies in the literature on the routine use and significance of CDM frozen sections. In the current study, we analyzed CDM frozen sections in all patients with resectable GBC regardless of tumor location.

In the present study, out of 14 patients with positive CDM, one patient had a tumor in the fundus/body region (distant from the cystic duct) and had CBD involvement in histopathological examination (HPE). This explains that the patient had a non-contiguous spread of the primary tumor to the cystic duct and/or EHBD. Shimizu et al. [17] described four patterns of spread to the HDL and showed that tumor spread in GBC might be non-contiguous (type 3 spread).

Pawlik et al. [10] conducted a study to analyze the incidence of residual disease during re-resection in IGBC. In patients with microscopically positive CDM, residual disease in the resected CBD was 42.1%. Another study by Nakata et al. [18] reported a 53.8% incidence of CBD infiltration in patients with cystic duct spread. Similarly, in the current study, seven patients (50%) of 14 patients with positive CDM frozen showed EHBD involvement on histopathology.

As reported in the literature, patients who required additional bile duct resection had significantly higher blood loss, longer duration of surgery and postoperative hospital stay, and increased risk of postoperative morbidity [19,20]. In the present study, positive CDM requiring EHBD resection had significantly higher intraoperative blood loss (375 vs. 200 ml, p=0.026) and longer hospital stay (11.5 vs. five days, p=0.002), while postoperative morbidity was comparable (p=0.722). 

According to several studies, a positive CDM in GBC significantly predicts OS [9,18]. There are two mechanisms to explain the poor prognosis of positive CDM. First, the cystic duct has a lymphatic network that leads to HDL lymph nodal spread. Second, cancer can disseminate to EHBD by superficial spread or the lumen of the cystic duct. Vega et al. [9] performed a retrospective study to assess the initial CDM status in IGBC as a prognostic factor. They concluded that patients with positive CDM had lower OS than patients with negative CDM. Positive CDM was strongly associated with CBD recurrence. They also stated that patients who underwent EHBD resection for positive margins had similar OS to patients with negative CDM. Nakata et al. [18] reported that the patients with cancer spreading to the cystic duct in GBC had significantly lower three- and five-year survival rates than patients without cancer spread to the cystic duct. In the present study, after PSM, median RFS (24 months vs. 12 months, p=0.30) and OS (24.5 months vs. 20 months, p= 0.417) were comparable between groups (negative CDM vs. positive CDM). These results suggest that resection margin status, TNM stage, and adjuvant chemotherapy affect survival outcomes in GBC rather than CDM status. 

The present study performed univariate and multivariate analyses to determine the predictive factors for CDM positivity. After multivariate analysis, a tumor in the neck region of the GB (diagnosed preoperatively or intraoperatively) was identified as an independent predictive factor for positive CDM. Based on this finding, the application of CDM frozen biopsy should be mandatory in resectable GBC without obstructive jaundice involving the neck region. Given the rare possibility of a positive CDM, it may be avoided in tumors involving the fundus/body of the GB.

According to the literature, the tumor location in GBC can significantly influence the outcome [14,21,22]. T2 tumors involving the hepatic side of the GB (T2b) are more aggressive and have a worse prognosis than T2 tumors involving the peritoneal side (T2a) [21]. Similarly, Leigh et al. [14] performed a retrospective study to determine the significance of the anatomical location of tumors in GBC for the outcome. They reported that compared to fundus/body tumors, neck tumors have a higher rate of preoperative jaundice, significantly more EHBD resection and bile duct involvement in HPE, a higher rate of PNI, a comparable TNM stage distribution, a significantly shorter OS, and thus significantly worse prognosis. Kurahara et al. [23] also concluded that GBC with neck involvement had a higher rate of PNI, a more significant number of positive lymph nodes, and a worse prognosis than fundus/body tumors. The present study found that GBC involving the neck had significantly higher levels of bilirubin and ALP. Neck tumors had a higher incidence of PNI (50% vs. 25%, p=0.021), a positive CDM requiring CBD excision (43.3% vs. 1.3%, p=0.001), and a CBD involvement (20% vs. 1.3%, p=0.002). Both groups had a comparable TNM stage distribution. Neck tumors had a significantly shorter RFS and OS (17 vs. 30 months, p=0.012, and 24 vs. 36 months, p=0.048, respectively). These results are consistent with the previous studies.

The current study has several limitations. First, this study was inherently limited due to its retrospective nature and associated biases. Second, the patients with positive CDM were relatively small and therefore at risk of being underpowered. Third, the study covers a long period of time in which the management protocol, including adjuvant chemotherapy (regimen), was changed. Therefore, future prospective studies with a larger cohort are needed to validate these results. Despite its limitations, this study illustrates the role of routine use and the significance of CDM frozen biopsy in GBC. It provides a subgroup analysis to determine the prognostic utility of tumor location.

## Conclusions

Routine use of frozen biopsy of the CDM in patients with resectable GBC without jaundice, regardless of tumor location, can be avoided. Its use can be selectively preferred in patients with GBC involving the neck since CDM positivity is only found in one in a hundred resectable non-neck tumors and tumor location is found to be an independent predictive factor for CDM positivity. However, further prospective trials with a larger cohort will provide rigid results. Positive CDM has comparable survival outcomes to negative CDM, providing a similar R0 resection rate and TNM stage. However, neck tumors have a worse prognosis than non-neck tumors.
